# Genetic Programs Driving Oncogenic Transformation: Lessons from In Vitro Models

**DOI:** 10.3390/ijms20246283

**Published:** 2019-12-12

**Authors:** Eros Di Giorgio, Harikrishnareddy Paluvai, Raffaella Picco, Claudio Brancolini

**Affiliations:** Department of Medicine, Università degli Studi di Udine, P.le Kolbe 4, 33100 Udine, Italy; eros.digiorgio@uniud.it (E.D.G.); hari.paluvai@student.unisi.it (H.P.); raf.picco@uniud.it (R.P.)

**Keywords:** *RAS*, *MYC*, *HDAC4*, *G0S2*, *DOCK4*, *SRPX* interferon, inflammation, TCGA

## Abstract

Cancer complexity relies on the intracellular pleiotropy of oncogenes/tumor suppressors and in the strong interplay between tumors and micro- and macro-environments. Here we followed a reductionist approach, by analyzing the transcriptional adaptations induced by three oncogenes (*RAS*, *MYC*, and *HDAC4*) in an isogenic transformation process. Common pathways, in place of common genes became dysregulated. From our analysis it emerges that, during the process of transformation, tumor cells cultured in vitro prime some signaling pathways suitable for coping with the blood supply restriction, metabolic adaptations, infiltration of immune cells, and for acquiring the morphological plasticity needed during the metastatic phase. Finally, we identified two signatures of genes commonly regulated by the three oncogenes that successfully predict the outcome of patients affected by different cancer types. These results emphasize that, in spite of the heterogeneous mutational burden among different cancers and even within the same tumor, some common hubs do exist. Their location, at the intersection of the various signaling pathways, makes a therapeutic approach exploitable.

## 1. Introduction

Cancer is a complex disease that arises through the accumulation of specific genetic lesions, affecting oncogenes and tumor suppressor genes. These lesions hijack transcriptional/epigenetic machineries to reprogram the gene expression profile of the cells [1]. The final goal is to sustain a robust proliferation, invasion, and the suppression of cell death programs. The tumorigenic process is the consequence of the adaptations that tumor cells must achieve to overcome different crises. Escaping senescence, suppressing apoptosis, resolving starvation from blood supply, impinging the metabolic circuits, and evading immune surveillance represent the major achieved adaptations [2].

Cancer cells are part of a complex ecosystem. Together with fibroblasts, endothelial and the immune cells constitute and organize the tumor microenvironment [3]. These cells establish different interactions that are transduced and maintained by interconnected networks of signals. To unveil this complexity, system biology approaches can be adopted to integrate genomic and epigenomic data collected from tumor biopsies [4,5]. Moreover, the initial assumption of considering cancer as the expansion of a monoclonal population has been recently replaced by the evidence obtained by single-cell sequencing [6]. In each cancer, several subclones can co-exist and can expand or contract as a function of their fitness [6].

Within this complexity, it is important to define the crucial events that can be target for a therapeutic intervention. The social impact of the tumorigenic process must prompt us to focus all efforts in unveiling the key oncogenic addictions for each different tumor type.

Historically, in vitro transformation models have provided the first approach to understand the tumorigenic process [7]. The identification of oncogenes and tumor suppressor genes and the demonstration of the cooperation among oncogenes represent the major accomplishments of these model systems. Traditionally, rodent cells have been largely used. However, transformation of murine cells and human cells greatly differ [8]. Human cells are more resistant to oncogenic transformation and require more events [9]. In vitro transformation approaches frequently adopt viral oncoproteins to switch off important tumor suppressor genes, such as RB and TP53. Nevertheless, transformation of human cells can also be obtained in absence of viral genes [10]. An innovative version of the in vitro transformation studies is represented by the generation of human organoids. Under a 3D growth condition and a selected microenvironment, these primary cells can be engineered in vitro, using CRISPR/Cas9 genome editing approaches, to recapitulate the key oncogenic lesions of the primary tumors [11].

Importantly, in vitro tumorigenic models must find validation in vivo. The resetting of the transcriptomic output represents the major tool through which cancer cells modulate their plasticity and adapt to the microenvironment. Hence, in this manuscript, we have compared the transcriptional profiles of three different isogenic models of in vitro transformation. Our goal is to demonstrate a correspondence between the in vitro transformation process and cancer development in patients. Moreover, we would like to define a minimal gene signature, shared among different transformation models, that could represent a useful tool to unveil the Achilles’ heel of many cancers.

## 2. Results

### 2.1. In Vitro Transformation of Human Fibroblasts Achieved by Different Oncogenes Leads to the Activation of Both Distinct and Common Genetic Programs

In vitro transformation of human cells requires a limited number of genetic changes [7]. Viral genes such as the SV40 early region, which encodes both the large T and small t oncoproteins, in combination with the catalytic subunit of the telomerase (hTERT) have been commonly used to drive the immortalization and to circumvent senescence of normal human fibroblasts and kidney cells [12]. The subsequent introduction of strong oncogenes such as *RAS* or *MYC* triggers the cellular transformation. These cells are characterized by an anchorage independent growth and by the ability to generate tumors when injected in immunocompromised mice [12,13].

**Figure 1:**
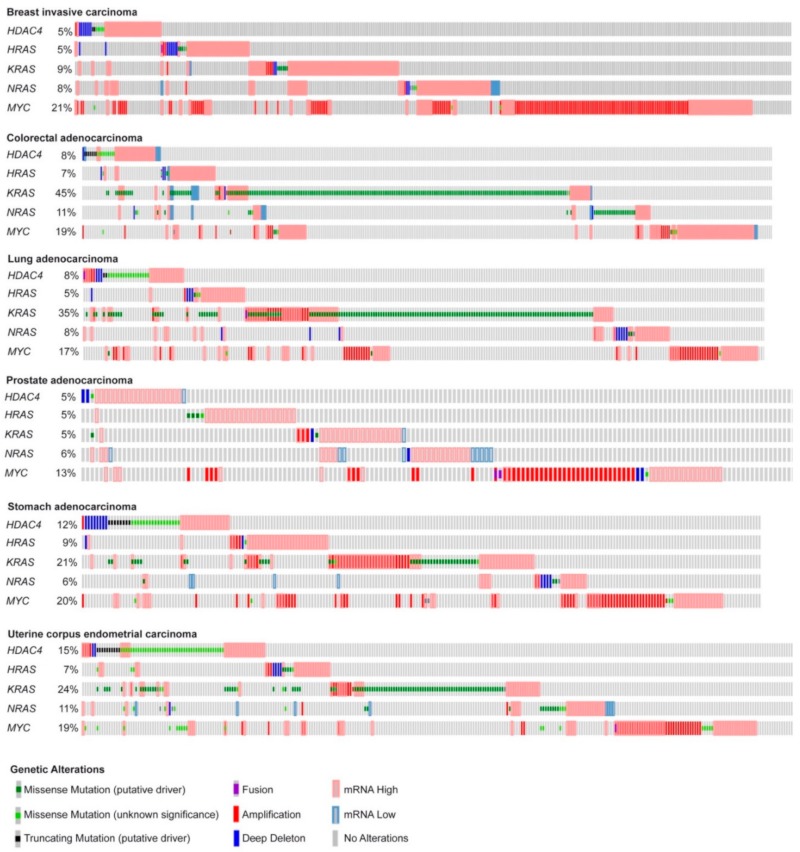
Summary of the genetic alterations reported for *HRAS*, *KRAS*, *NRAS*, *MYC,* and *HDAC4* in some human cancers. Oncoprints of *HDAC4*, *HRAS*, *KRAS*, *NRAS*, and *MYC* mutations and alterations in the indicated tumor types. Images were cropped to highlight the alterations. Data were obtained from the TCGA database. The heatmap was generated through cBioPortal (http://www.cbioportal.org). The different genetic alterations are indicated by the provided color code.

Here, we deeply investigated the strength of the transcriptome reprogramming, triggered during the in vitro transformation process, in predicting the outcome of different human cancers. With this aim we compared the gene expression profiles of human foreskin fibroblasts expressing *hTERT*, the early region of SV40 and subsequently transduced with *HRAS/G12V* [13], *MYC* [14], or an oncogenic nuclear resident form of *HDAC4* (*HDAC4-TM*) [13,15]. These oncogenes have been chosen for their heterogeneity. As frequently reported for other oncogenic combinations [12,13], when mutations, amplifications, or transcript dysregulations of these oncogenes were analyzed in different human cancers, the co-occurrence of their alterations appears to be restricted to a small number of cases. In Figure 1, we report the oncoprint of TCGA PanCancer ATLAS datasets selected among some of the most frequent solid tumors. We included breast, colorectal, lung, prostate, stomach, and uterine cancers (Figure 1). As expected, point mutations in *RAS* are not common in breast and prostate cancer [16]. Interestingly, genetic alterations in *HDAC4* are more frequent in uterine and stomach cancers, with a conspicuous incidence of truncations and point mutations of still unknown impact on the activities of this deacetylase (Figure 1).

The simultaneous dysregulations of *HDAC4*, *MYC*, and *RAS* oncogenes are relatively rare. There are some co-occurrences between *HDAC4* and *MYC* or *HDAC4* and *RAS* as well as between *MYC* and RAS. Most frequently, patients carry alterations only in one of the three oncogenes (Figure 1). This evidence suggests that the three oncogenes can act through completely distinct and non-complementary mechanisms or through at least partially overlapping pathways.

### 2.2. RAS, MYC, and HDAC4-Mediated Oncogenic Transformation Is Marked by the Dysregulation of Common Pathways Rather Than of Common Genes.

To prove the above enounced concept, we interrogated the gene expression profiles of BJ-*hTERT/ST/LT/MYC* [14], BJ-h*TERT/ST/LT/HRASG12V* [13], and BJ-*hTERT/ST/LT/HDAC4-S246A, S467A, S632A* [15], relatively to the isogenic pre-transformed control cells, expressing the SV40 LT and ST or the entire early region.

By adopting as cut-off criteria 1.5-fold change and FDR < 0.05, we obtained six signatures of genes regulated by the three oncogenes. Overall, 519 and 634 transcripts were respectively upregulated and downregulated by MYC, 556 and 595 by RAS and finally, 551 and 979 by HDAC4.

**Figure 2:**
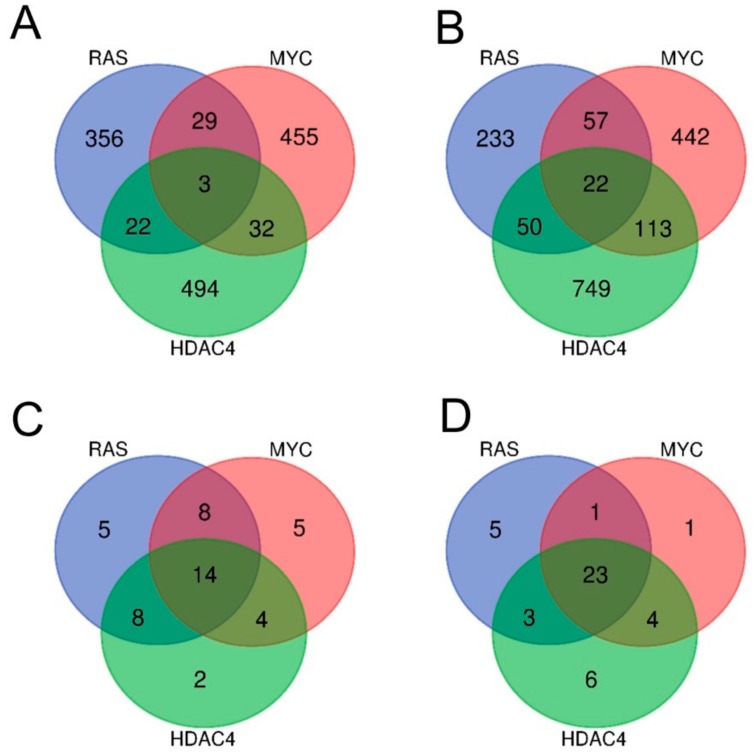
Analysis of the transcriptional profiles in three different models of in vitro transformation. (**A**) Venn diagram showing the number of transcripts upregulated during the transformation process in BJ/*hTERT*/*LT*/*ST* cells expressing RAS, MYC, or HDAC4 as indicated. (**B**) Venn diagram showing the number of transcripts downregulated during the transformation process in BJ/*hTERT/LT/ST* cells expressing RAS, MYC, or HDAC4 as indicated. (**C**) Venn diagram showing the number of different hallmarks gene sets significantly upregulated by RAS, MYC, and HDAC4. (**D**) Venn diagram showing the number of different hallmarks gene sets significantly downregulated by RAS, MYC, and HDAC4. All the Venn diagrams were created by using this software http://bioinformatics.psb.ugent.be/webtools/Venn/.

Few genes turned out to be commonly dysregulated by the three oncogenes. Only three genes were upregulated either in RAS, MYC, and HDAC4 transformed cells (Figure 2A), while 22 were the commonly repressed genes (Figure 2B).

Although the commonly dysregulated genes are rare, the three oncogenes could influence the same pathways through alterations of different genes, operating at different steps of the same pathway. To prove this hypothesis, we applied the Gene Set Enrichment Analysis (GSEA) algorithm to compare the six signatures obtained to the MSigDB HALLMARK gene sets [17,18]. The “HALLMARKS” is a collection of 50 gene sets; each of them groups genes that display coordinate expression and represent well-defined biological processes. Tables SIA–SIC summarize the statistically significant genes sets identified by the analysis for the upregulated genes and Tables SIIA–SIIC for the downregulated genes. Venn diagrams show that the three oncogenes share several gene sets, which control important biological functions related to the transformation process. For the upregulated genes, 14 different gene sets were commonly regulated by RAS, MYC, and HDAC4 (Figure 2C). For the repressed genes this number increased up to 23 (Figure 2D).

In summary, although the number of genes commonly regulated in the three models of in vitro transformation is small, the pathways and the biological processes under the influences of HDAC4, RAS, and MYC testify a convergence towards common strategies of hijacking specific cellular responses.

### 2.3. Identification of the Pathway Reprogramming Core that Defines the Common Trait of the in Vitro Transformation Process

Having found that the three transformation models influence common gene sets, we next analyzed which genes were under the regulation of the three oncogenes in the different gene sets.

**Figure 3:**
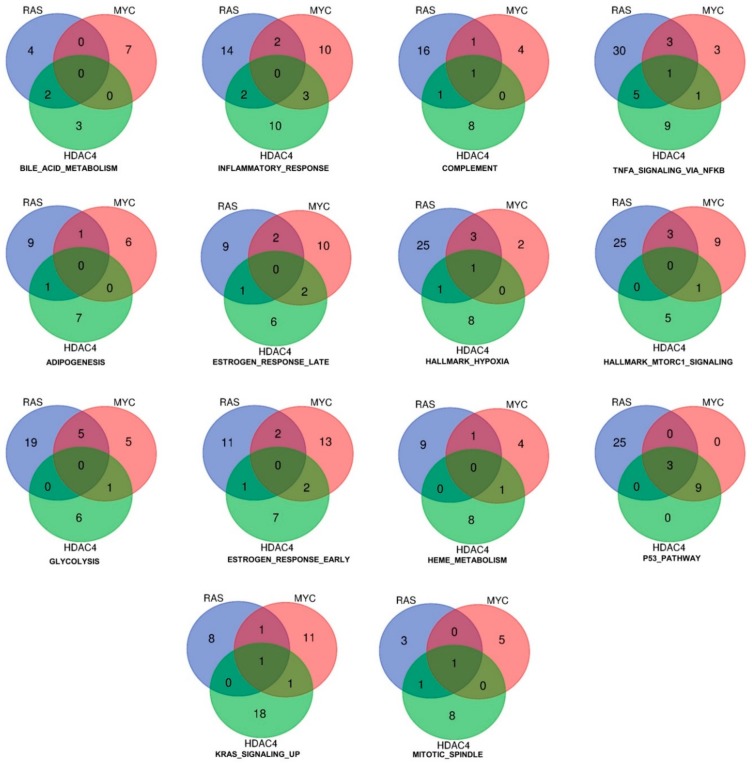
Hallmarks and genes commonly upregulated by the three oncogenes (HDAC4, RAS, MYC) during the in vitro transformation process. Venn diagram showing different sets of hallmarks and the number of upregulated genes, which are shared by HDAC4, RAS, and MYC.

Figure 3 shows the result of such analysis for the upregulated genes. As above mentioned, only three genes (*DOCK4*, *G0S2*, *SRPX*) were commonly induced by the three oncogenes. These genes are represented in more than one gene set. The Venn diagrams illustrate also the common genes among two models of in vitro transformation (Figure 3). Interestingly, the gene set “p53 pathway” includes all the three genes. Although no other genes were in common between RAS and MYC or RAS and HDAC4, all genes (*n* = 9) were in common between MYC and HDAC4. This observation suggests that HDAC4 and MYC share similar strategies to dysregulate the p53 pathway. Another relevant gene set is the “inflammatory response”. Here common genes were not identified. However, this cellular response is equally modulated by all three oncogenes, with a similar number of genes. Some of them are in common between RAS and HDAC4 (*n* = 2), RAS and MYC (*n* = 2), and MYC and HDAC4 (*n* = 3). In the case of the gene set “glycolysis” several genes are under the influence of RAS and a good overlap is observed with MYC (*n* = 5). By contrast, HDAC4 shows a peculiar influence on this metabolic gene set, possibly reflecting its non-conventional activities on the metabolism of the transformed cells [19].

**Figure 4:**
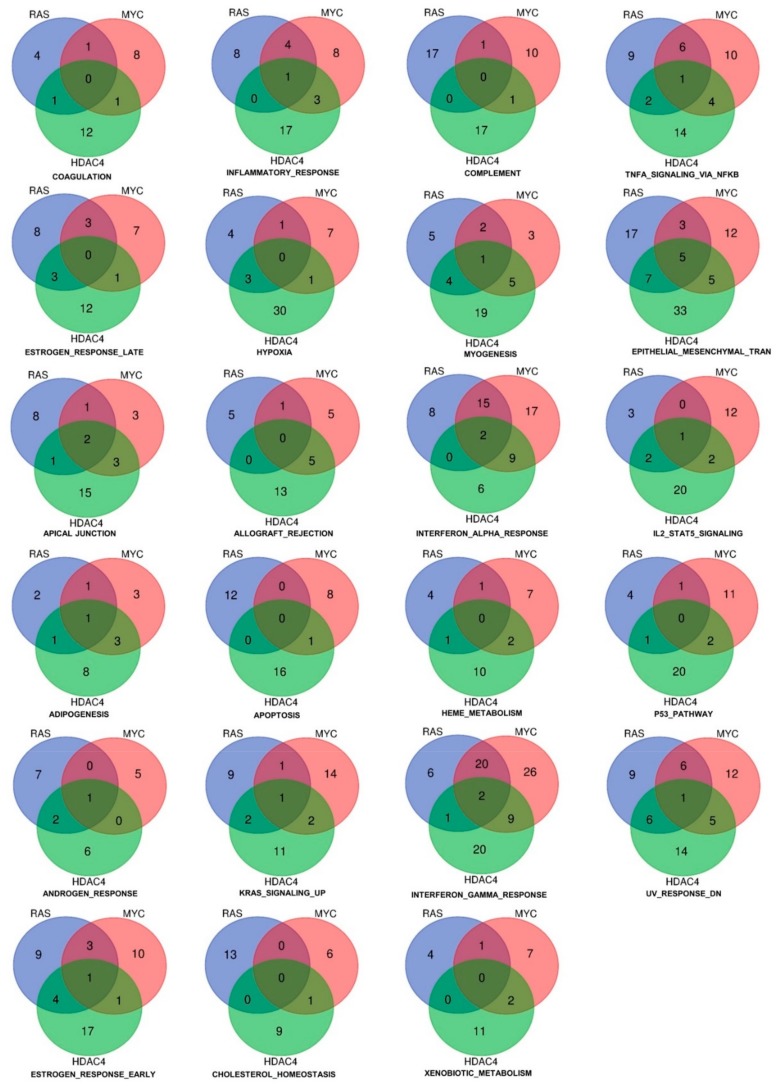
Hallmarks and genes commonly downregulated by three oncogenes (HDAC4, RAS, MYC) during the in vitro transformation process. Venn diagram showing different sets of hallmarks and number of downregulated genes that were commonly shared by HDAC4, RAS, and MYC.

When we analyzed the repressed genes, the number of statistically significant hallmarks gene sets was higher (*n* = 23) (Figure 4 and Tables SIIA–SIIC). The gene set “epithelial-mesenchymal transition” scored the highest number of common hubs (*n* = 5). In addition, a significant number of genes were similarly dysregulated by RAS and HDAC4 (*n* = 7) and by MYC and HDAC4 (*n* = 5). The EMT gene set was the most statistically significant enriched gene set in the RAS and HDAC4 transformed cells, whereas in MYC-transformed it scored the fourth position. An opposite behavior was observed for the gene set “complement”. The total number of genes regulated was 46 but only 2 genes were in common between RAS and MYC and HDAC4 and MYC. This observation indicates that the complement pathway is targeted through alternative mechanisms in the three models of in vitro transformation.

Other statistically significant repressed gene sets are the *interferon-α* (IFNα) and the *interferon-γ* (IFNγ) responses. In RAS-transformed cells they resulted the second and the third top hits, respectively, whereas in MYC-transformed cells the second and the first. Finally, in the case of HDAC4 they scored as the seventh and the third hits. 

Only three genes were commonly repressed in the IFNs hallmarks gene sets: *ARID5B*, *ELF1*, and *MX1*. The last was present in both the IFNα and the IFNγ gene sets. When RAS and MYC were compared, 20 genes were in common for the IFNγ response and 15 in the case of the IFNα. On the other side, *HDAC4* shows stronger similarities with *MYC*. Nine genes belonging to IFNγ and to IFNα gene sets were in common between MYC and HDAC4 in IFNγ as well as in IFNα gene sets. Few similarities were found between *HDAC4* and *RAS*, with only a gene in common for IFNγ (Figure 4).

In summary, this analysis indicates the IFNα and IFNγ signaling are significantly and robustly repressed in different models of in vitro transformation. This can be due to: (i) the key role that the repression of the pathways plays in the transformation process, as previously reported [20,21,22,23,24]; (ii) a certain degree of purifying selection that supervise the conservation of IFN signaling [25]. 

### 2.4. Definition of the Minimal Signatures Regulated during in Vitro Transformation

The final goal of our approach is to verify if the pathways dysregulated during the in vitro transformation can predict the outcome of malignant cancers in patients. For this reason, we extracted three signatures: Two for the repressed and one for upregulated genes (Table 1). Both signatures are made up of genes commonly and significantly upregulated or downregulated by the three oncogenes.

The signature of the upregulated genes groups *DOCK4*, *G0S2*, and *SRPX* (Table 1). *DOCK4* encodes for a guanine nucleotide exchange factors that participates in the regulation of cell adhesion and membrane trafficking [26,27]. It is reported to be mutated in cancer [26] and to influence cancer aggressiveness through the modulation of WNT and TGF-β pathways [28,29]. Since also anti-proliferative activities have been reported, its oncogenic potential seems to be context specific [30,31]. 

*G0S2* (G0/G1 switch gene 2) encodes for a potent inhibitor of adipose triglyceride lipase. In this manner, G0S2 acts as a master regulator of the tissue-specific balance of triglyceride storage vs. mobilization [32]. Correlations with cancer are unclear with anti-proliferative effects described by some reports [33,34,35]. Finally, *SRPX* (sushi repeat containing protein X-linked), known also as *ETX1* or *DRS*, was initially isolated as deleted in patients with X-linked retinitis pigmentosa [36], as well as downregulated by *v-src* [37]. A tumor suppressive activity for *SRPX* was proposed [38]. Moreover, its expression seems to be downregulated in different aggressive cancers [39,40,41].

For the repressed genes we selected two different signatures. The first signature (repressed signature A) includes five genes (*CDH11*, *DKK1*, *GREM1*, *MYLK*, *SPRY2*). These genes do not belong to the inflammatory and interferon gene sets (Table 1). A second signature of genes (repressed signature B) groups the remaining commonly repressed genes that belong to inflammatory-immune responses (*ARID5B*, *DUSP4*, *ELF1*, *LPAR1*, *MX1*, *SOCS2*, *TNFRSF11B*).

### 2.5. High mRNA Levels of the Upregulated Genes Correlate with Reduced Patients’ Survival in a Group of Different Tumors

In order to understand if the identified signatures can predict cancer aggressiveness and patients’ outcome, they were used to interrogate the survival data of 32 cancer types deposited in the Cancer Genome Atlas (TCGA).

**Figure 5:**
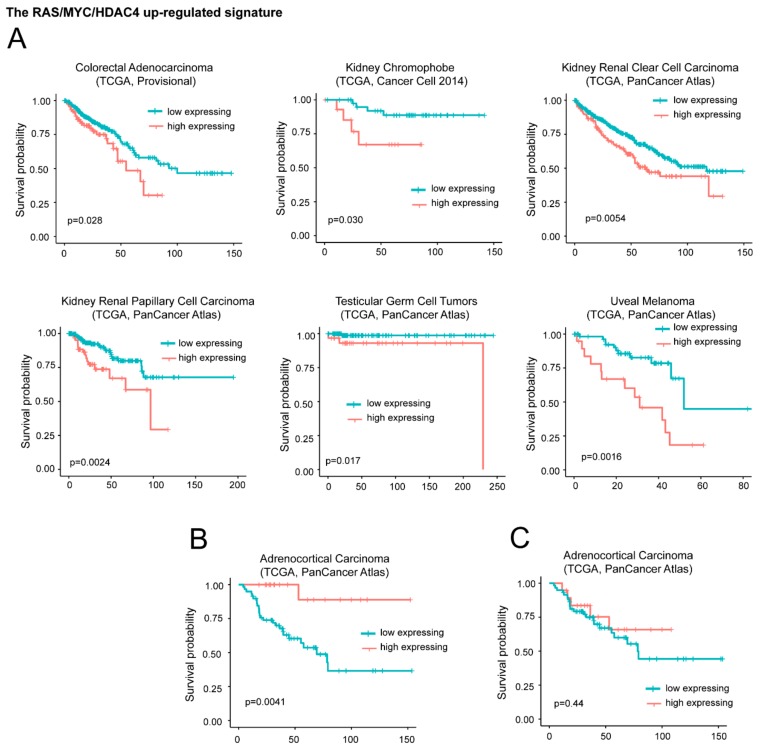
High mRNA levels of upregulated genes during in vitro transformation process influence patients’ survival. (**A**) Kaplan-Meier survival analysis related to the three upregulated genes *DOCK4*, *G0S2*, *SRPX.* All cases were analyzed and clustered into two groups according to *DOCK4*, *G0S2*, and *SRPX* expression levels. High levels of expression group (> the third quartile/high expressing) compared to all other cases (< the third quartile/low expressing). Cases were: Colorectal adenocarcinoma (all *n* = 382, high expressing *n* = 95), kidney chromophobe (all *n* = 65, high expressing *n* = 16), kidney renal clear cell carcinoma (all *n* = 510, high expressing *n* = 127), kidney renal papillary cell carcinoma (all *n* = 283, high expressing *n* = 70), testicular germ cell tumors (all *n* = 149, high expressing *n* = 37), and uveal melanoma (all *n* = 80, high expressing *n* = 20). (**B**) Kaplan-Meier survival analysis related to *G0S2* in adrenocortical carcinoma (ACC). High level expression (above the third quartile) of *G0S2* in ACC were observed in 19 cases. All cases were *n* = 78. (**C**) Kaplan-Meier survival analysis related to *DOCK4* and *SRPX* in ACC. High level expression (> the third quartile) of *DOCK4* and *SRPX* were observed in 19 cases, all cases were *n* = 78.

For the upregulated genes, we segregated the patients in two groups. The first group is characterized by high expression levels of the signature (above the third quartile). The second group is characterized by moderated upregulation, unperturbed or repressed expression of the signature (below the third quartile). Figure 5A illustrates that in six different cancer types (colorectal adenocarcinoma, kidney chromophobe, kidney renal clear cell carcinoma, kidney renal papillary cell carcinoma, testicular germ cell tumors, and uveal melanoma) high levels of *DOCK4*, *G0S2*, and *SRPX* significantly correlate with a worst survival.

On the opposite high levels of the signature predicts a better overall survival in adrenocortical carcinoma (ACC) (Figure 5B). This behavior could depend on *G0S2*. In fact in ACC, which is a rare, aggressive malignancy, *G0S2* hypermethylation is a hallmark of rapidly recurrent or fatal disease, amenable to targeted assessment using routine molecular diagnostics [35]. Very low levels of *G0S2* mRNA expression characterize tumors with *G0S2* hypermethylation. Although low *G0S2* expression marks 40% of ACC and independently predicts shorter disease-free and overall survival, the role of this gene in adrenocortical biology is still unknown [42]. To confirm this data, we repeated the survival analysis without the *G0S2* gene. In this case the positive correlation with ACC survival was abrogated (Figure 5C). For the remaining 25 cancer types the signature failed to predict any patients’ survival.

### 2.6. High Levels of DOCK4, G0S2, and SRPX Expression Are Related to Worse Survival in Similar but also Different Tumor Types

**Figure 6:**
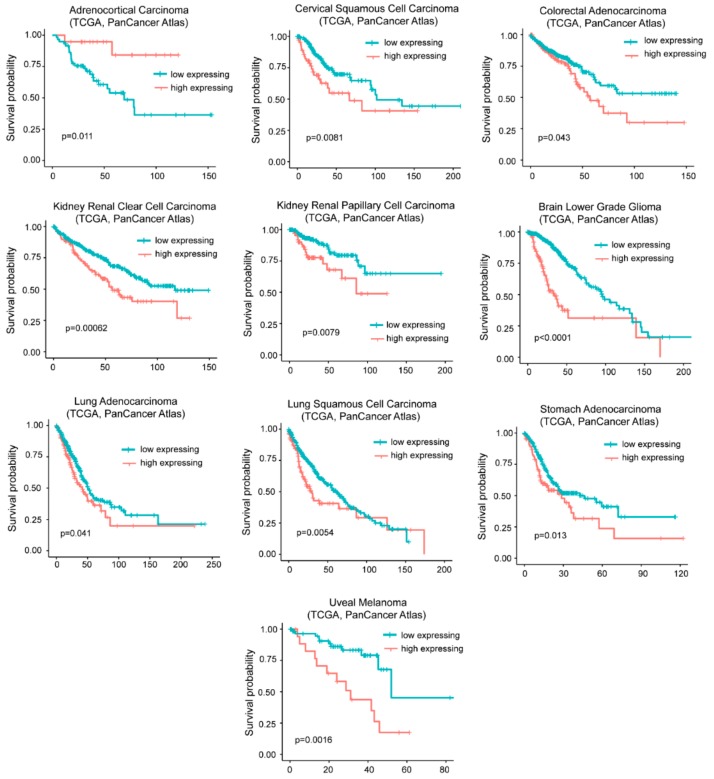
High levels of *G0S2* expression correlate with reduced survival in different cancer types. TCGA survival data analysis on tumors grouped for high levels of *G0S2* expression alone (> the third quartile/high expressing) compared to all other cases (< the third quartile/low expressing). Cases were: Adrenocortical carcinoma (all *n* = 78, high expressing *n* = 19), cervical squamous cell carcinoma (all *n* = 294, high expressing *n* = 73), colorectal adenocarcinoma (all *n* = 592, high expressing *n* = 148), kidney renal clear cell carcinoma (all *n*=510, high expressing *n*=127), kidney renal papillary cell carcinoma (all *n* = 283, high expressing *n* = 70), brain low grade glioma (all *n* = 514, high expressing *n* = 128), lung adenocarcinoma (all *n* = 510, high expressing *n* = 127), lung squamous cell carcinoma (all *n* = 484, high expressing *n* = 121), stomach carcinoma (all *n* = 412, high expressing *n* = 103), and uveal melanoma (all *n* = 80, high expressing *n* = 20).

The influence of *G0S2* methylation on cancer mortality is peculiar of ACC. As expected from the in vitro transformation models, in many other tumors (*n* = 9) high levels of *G0S2* mRNA are indicative of a reduced survival (Figure 6).

To verify whether *G0S2* is the key gene in predicting patients’ survival, we repeated the analysis for *DOCK4* and *SRPX*. High levels of *DOCK4* correlates with a reduced survival in five different tumors (colorectal, kidney chromophobe, brain low grade glioma, stomach adenocarcinoma, and uveal melanoma) and with a better prognosis in two cancer types (mesothelioma and skin cutaneous melanoma) (Figure S1). Increased levels of *SRPX* correlates with an increased hazardous rate in seven different cancer types (bladder, colorectal, head and neck squamous carcinoma, kidney renal clear cell and papillary carcinomas, thyroid carcinoma, and uterus corpus endometrial carcinoma). Conversely, it predicts a better outcome in melanoma (Figure S2). This analysis suggests that the three upregulated genes could exert independent activities to influence cancer aggressiveness.

### 2.7. A Group of Genes Repressed during in Vitro Transformation Predicts Patients’ Survival.

Next, we interrogated the TCGA survival data with the repressed signature A. The patients were segregated in two groups accordingly to the median value of expression of the signature. In two different cancer types, high levels of the signature correlate with increased survival, while in other six cancer types high levels of the same signature correlate with reduced survival. Hence, we discarded this signature for further analysis.

**Figure 7:**
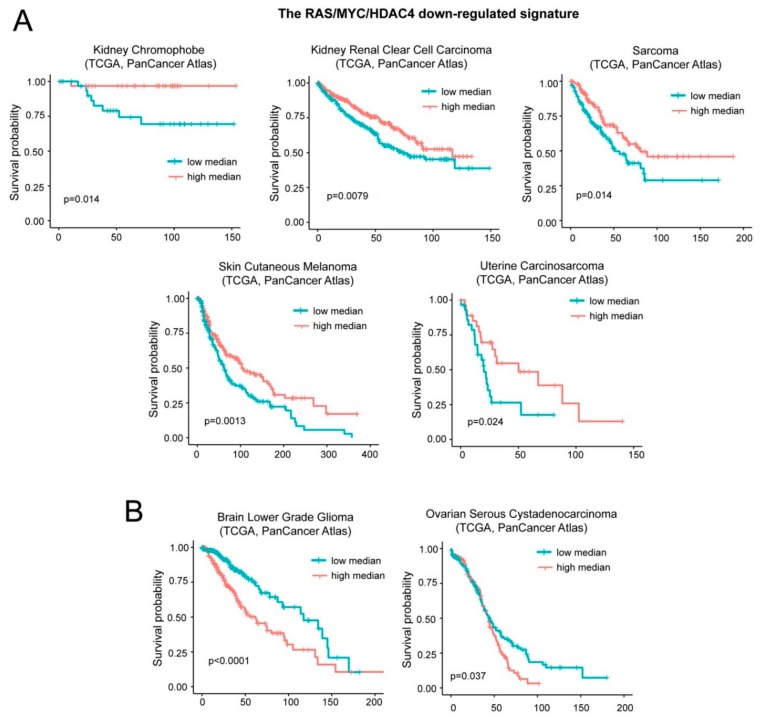
Patients’ survival and expression levels of oncogene-repressed inflammatory signature. (**A**) Kaplan-Meier survival analysis related to the oncogene-repressed inflammatory signature. All cases were analyzed and clustered into two groups according to median expression levels (high median; expression levels > the median and low median; expression levels< the median). Cases were: Kidney chromophobe (all *n* = 65, high expressing *n* = 32), kidney renal clear cell carcinoma (all *n* = 510, high expressing *n* = 255), sarcoma (all *n* = 253, high expressing *n* = 126), skin cutaneous melanoma (all *n* = 443, high expressing *n* = 221), and uterine carcinosarcoma (all *n* = 57, high expressing *n* = 28). (**B**) Kaplan-Meier survival analysis related to the oncogene-repressed inflammatory signature. All cases were analyzed and clustered into two groups according to median expression levels (high median; expression levels > the median and low median; expression levels < the median). Cases were: Brain low grade gliomas (all *n* = 514, high expressing *n* = 257) and ovarian serous cystadenocarcinoma (all *n* = 300, high expressing *n* = 150).

A different result was obtained with the repressed signature B that groups inflammatory and interferon genes. Low levels of the repressed signature correlate with a worst prognosis in five cases: Kidney chromophobe carcinoma, kidney clear cell carcinoma, sarcoma, skin cutaneous melanoma, and uterine carcinosarcoma (Figure 7A). On the opposite, low levels of this signature predict a better outcome in patients with brain lower grade glioma and ovarian serous cystadenocarcinoma (Figure 7B). For simplicity we named this signature “oncogene-repressed inflammatory signature”.

### 2.8. Inflammatory Genes Repressed during the in Vitro Transformation Process and Tissue-Infiltrating Immune Cells

Tumors are characterized by a variegated abundance of tissue infiltrating immune cells, which can influence the prognosis [43]. Since the oncogene-repressed inflammatory signature comprises many inflammatory genes, our in vivo analysis could be influenced by the presence of tumor infiltrating immune cells. Recent studies have shown that the abundance of immune cells in tissue transcriptomic data can be predicted by specific mRNA signatures [44].

We applied the microenvironment cell populations-counter (MCP-counter) to evaluate the contribution of different immune cells to the survival outcomes of patients analyzed for the oncogene-repressed inflammatory signature. With the exclusion of kidney chromophobe, uterine carcinosarcoma, and ovarian serous cystadenocarcinoma, in the other cancer types the survival was also influenced by the presence of immune cells, although with different outcomes (Figures S3 and S4). Prognosis of skin cutaneous melanoma, sarcoma, and brain low grade glioma (LGG) is influenced by the infiltration of different subtypes of immune cells. These tumors behave differentially, with only LGG showing a worst outcome. On the contrary, the better outcome of kidney renal cell carcinoma results significantly and specifically associated with the presence of myeloid dendritic cells (Figure S4).

In conclusion, although in some tumor types the repression of the inflammatory signature and the resulting reduced patients’ survival can be specifically ascribed to the transcriptional reprogramming of the neoplastic cells; in other tumors, we cannot exclude a contribution of the immune cells. These cells may have been isolated along with the tumor tissue and thus contribute to the transcriptional landscape as reported by the TCGA data.

### 2.9. Oncogene-Repressed Inflammatory Signature Provides an Additional Contribution to Overall Survival

As a final analysis, we decided to evaluate the impact on patients’ survival of both the tumor infiltrating immune cells and of the expression levels of the oncogene-repressed inflammatory signature. The analysis was restricted to tumor types that showed significant correlations between the overall survival and the presence of the immune cell infiltrates (Figures S3 and S4). 

**Figure 8:**
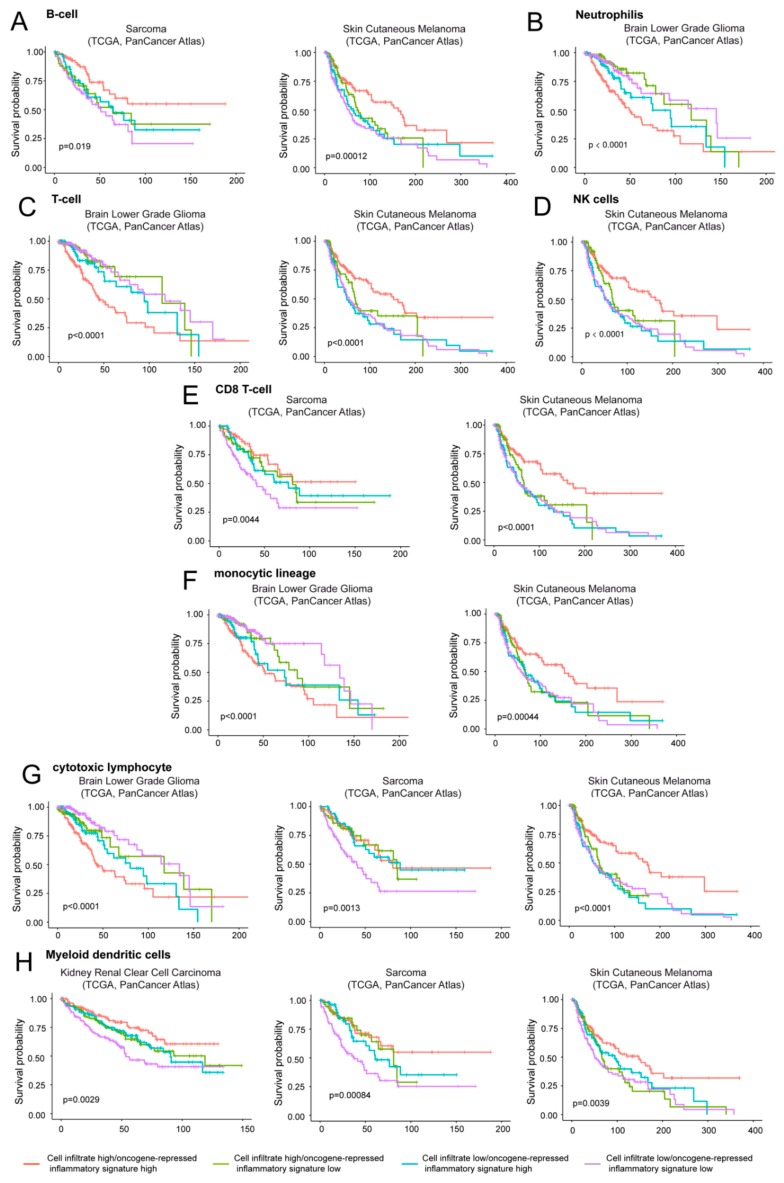
The contribution of infiltrating immune/inflammatory cells to overall survival. Kaplan-Meier survival analysis related to the immune/inflammatory signature and the infiltration of different immune/inflammatory cells. Infiltrating immune/inflammatory cells were defined as described in materials and methods. Patients were grouped high/high (high levels of both infiltrating cells and immune signature), high/low (high levels of infiltrating cells and low levels of the oncogene-repressed inflammatory signature), low/high (low levels of infiltrating cells and high levels of the oncogene-repressed inflammatory signature), and as low/low (low levels of both infiltrating cells and of the oncogene-repressed inflammatory signature). The four groups were generated according to median expression levels of the two signatures. Cases: (**A**) Sarcoma (all = 253, high/high = 75, high/low = 51, low/high = 51, low/low = 76) and skin cutaneous melanoma (all = 428, high/high = 133, high/low = 82, low/high = 82, low/low = 131). (**B**) Brain low grade glioma (all = 512, high/high = 155, high/low = 102, low/high = 101, low/low = 154). (**C**) Brain low grade glioma (all = 512, high/high = 164, high/low = 92, low/high = 92, low/low = 164) and skin cutaneous melanoma (all = 428, high/high = 148, high/low = 66, low/high = 67, low/low = 147). (**D**) Skin cutaneous melanoma (all = 428, high/high = 143, high/low = 72, low/high = 72, low/low = 141). (**E**) Sarcoma (all = 253, high/high = 70, high/low = 56, low/high = 56, low/low = 71) and skin cutaneous melanoma (all = 428, high/high = 136, high/low = 77, low/high = 79, low/low = 136). (**F**) Brain low grade glioma (all = 512, high/high = 164, high/low = 93, low/high = 92, low/low = 163) and skin cutaneous melanoma (all = 428, high/high = 140, high/low = 73, low/high = 75, low/low = 140). (**G**) Brain low grade glioma (all = 512, high/high = 153, high/low = 104, low/high = 103, low/low = 152), sarcoma (all = 253, high/high = 74, high/low = 52, low/high = 52, low/low = 75) and skin cutaneous melanoma (all = 428, high/high = 142, high/low = 74, low/high = 73, low/low = 139). (**H**) Kidney renal clear cell carcinoma (all = 510, high/high = 132, high/low = 123, low/high = 123, low/low = 132), sarcoma (all = 253, high/high = 70, high/low = 56, low/high = 56, low/low = 71) and skin cutaneous melanoma (all = 428, high/high = 126, high/low = 89, low/high = 89, low/low = 124).

The three tumor types analyzed were LGG, skin cutaneous melanoma, and sarcoma (Figure 8). In LGG, the coexistence of high levels of expression of the oncogene-repressed inflammatory signature and the presence of immune cells, particularly of T cells, cytotoxic lymphocytes and neutrophils represent a negative prognostic condition (Figure 8B,C,G). In agreement with our data, strong correlations between the risk score and T cells, macrophage-related immune response, as well as the expression of immunomodulators were recently reported in LGGs [3,45].

Interestingly, in sarcoma and melanoma tumors where high levels of the oncogene-repressed inflammatory signature correlate with an increased survival, two different behaviors can be appreciated. In sarcoma the presence of immune cells is dominant. In fact, when immune cell infiltrates (particularly CD8 T cells and cytotoxic lymphocytes) are observed, increased survival is maintained, also in the presence of low levels of the oncogene-repressed inflammatory signature (Figure 8E,G). In contrast, in skin cutaneous melanoma low expression levels of the signature are sufficient to reduce the overall survival (Figure 8A,C,D,F,G). In melanoma, the best prognosis is observed in the presence of high expression of the oncogene-repressed inflammatory signature and the concomitant presence of immune cells, particularly T cells, NK, CD8 T cells, and cytotoxic lymphocytes.

## 3. Discussion

In this manuscript, we compared three isogenic models of in vitro transformation and we identified minimal signatures that characterize the transformation process. We figured out that most of the oncogenic programs driven by the three oncogenes rely on the activation of common pathways. Curiously, these pathways seem to be activated through alternative/complimentary mechanisms, as the modulated transcripts deeply differ and overlap only partially. This could be expected as RAS and MYC in cancer establish a cooperation based on the integration rather than on the intersection of their genetic programs [46]. Moreover, this evidence suggests that the third selected oncogene, HDAC4, triggers a different transformation process, as suggested previously [19,47,48,49,50]. However, even if through alternative roads, the three oncogenes converge on some common hubs [2]. 

A minimal signature of three upregulated transcripts identified through our analysis successfully predict worse prognosis in some cancers. Among these genes emerges *G0S2*. This inhibitor of adipose triglyceride lipase governs lipolysis and fatty acid (FA) availability. G0S2 is abundantly expressed in adipose tissue and *G0S2* transgenic mice experience difficulties in shifting from carbohydrate to FA oxidation during fasting [51]. In vivo studies have indicated that G0S2 could ensure the usage of glycogen-derived glucose as the primary source of rapid energy output [32,51]. This influence could be relevant for the metabolic adaptation of cancer cells. However, preliminary studies have provided conflicting data on a role of *G0S2* in cancer cells [34,52,53,54]. Our discovery about the existence of strong correlations between *G0S2* levels and patients’ survival in different cancers suggests the needing of a more extensive investigation about the impact of this gene on the metabolism and proliferation of transformed cells. 

Repression of interferon-inducible genes is a well-known feature of the RAS-dependent transformation process [55,56,57,58,59]. Similarly, correlations between MYC and interferon have been known since a long time. Initially, it was discovered that IFNs can regulate MYC expression [60,61]. Subsequently, a suppressive activity of MYC on IFN signaling was reported [62]. This repressive influence was further proved by transcriptomic studies [63,64,65,66,67]. The recent discovery that the targeting of MYC through an epigenetic therapy provides an important advantage for an efficient immunotherapy could represent an important clinical perspective of all these studies [68,69].

Our results justify the inclusion of HDAC4 in the group that comprises two historically oncogenes for being similarly able to repress IFN genes during the transformation process. In the in vitro model of transformation, the presence of SV40 genes—which promote the expression of the interferon response [70]—could overestimate the repressive influence of the cellular oncogenes on this pathway. Nevertheless, alterations of the interferon genes were observed also in vivo and independently from the presence of the immune cells (Figure 8). Switching off the IFN and the inflammatory responses could provide a double advantage to the transformed cells, both in a cell autonomous and non-autonomous manner. It can favor the transformation process, by limiting tumor suppressive actions (such as apoptosis), and it can influence the tumor microenvironment and the immune response [71]. In vivo experiments on murine models of cancer have clarified that, in the initial tumorigenic steps, a strong inflammatory environment is promoted by the cancer cells themselves [72]. The release of chemokines, cytokines, and growth factors, as a consequence of the DNA damage accumulated during the early transformation process, promotes the infiltration and proliferation of immune cells that set up the first line of anti-cancer extracellular responses [73]. When full transformation is achieved, cancer cells drive a strong anti-inflammatory response, through intracellular clues—such as the activation of the IL10-Stat3 pathway and the release of extracellular molecules—which recall immune suppressor cells [74]. The balance of the anti-tumor response is then further compromised by the effect of the stroma and of the microenvironment, with the involvements of cancer associated fibroblasts (CAFs) and of tumor associated macrophages (TAMs). 

It is curious that these two steps are exactly recapitulated during the in vitro transformation, with the arising of a type I IFN response during oncogene induced senescence or in the steps that come immediately before the full oncogenic conversion. After this step, the anti-inflammatory responses become predominant, as examined in this manuscript. The concept that some of these key features are triggered also in cancer cells cultured in petri dish suggests that the microenvironment acts by sculpting pathways that are already well established and poised in tumor cells in face of intracellular survival needs [75].

In conclusion, our analysis evidences that different oncogenes use common pathways to reach malignancy and to set up a barrier against the immune aggression.

## 4. Materials and Methods

### 4.1. Data Retrieval and Analysis

The transcriptional profiles of the three isogenic transformation models were obtained by re-analyzing the datasets GSE17941 [13], GSE72530 [14], and GSE120040 [15] deposited as raw files in GEO (Gene Expression Omnibus). The CEL files were processed with affy package in R [76]. Multiple callings coming from redundant probes were reduced to a single signal per gene by using Unigene ID centered CDFs (Chip Description Files) retrieved from the Molecular and Behavioral Neuroscience Institute Microarray Lab (URL: http://brainarray.mbni.med.umich.edu/Brainarray/Database/CustomCDF/genomic_curated_CDF.asp) [77]. RMA algorithm was used for normalization [78]. In the three datasets selected, the hybridization was done on three different Affymetrix chips (platforms HG-U133A_2, HuGene-1_0-st-v1 and Clariom_S). For the identification of differentially expressed genes (DEGs), the limma package [79] was used. The calling of significance was based on a 1.5-fold change/FDR < 0.05 criteria. In each dataset, the transformation model represented by pre-transformed BJ cells expressing RAS G12V (GSE17941) or MYC (GSE72530) or HDAC4 (GSE120040) was compared to the pre-transformation model which is represented by BJ fibroblasts expressing hTERT, LT, and ST SV40 genes. 

### 4.2. Enrichment Analysis

The “HALLMARK” collection of 50 gene sets deposited in the Molecular Signatures Database (MSigDB) (subject) was interrogated with the three DEG lists generated (query). The MSigDB analysis tool (Broad Institute (http://www.broadinstitute.org/gsea/msigdb/index.jsp) algorithm was used to score the overlap between queries and subjects. The identified hits were ranked by the enrichment score and the *p*-value (FDR < 0.05) [17,18].

### 4.3. Generation of the Signatures of Transformation

The signature of the induced genes includes three genes that were significantly upregulated in the three selected models of transformation. The signature of the repressed genes includes 13 genes commonly downregulated in the three models of transformation. This signature was further sub-divided in sub-signatures A and B, where A includes six genes that do not belong to the HALLMARK gene sets “inflammation” and “interferon response” and B includes the other seven genes. 

### 4.4. Analysis of Survival

mRNA expression data coming from RNAseq studies and normalized by the expectation-maximization (RSEM) method and patients’ clinical data about 32 cancer studies were retrieved from TCGA, using the R package cgdsr [80]. Hits corresponding to patients with incomplete expression or survival data were discarded. The final created dataset groups 11,424 samples belonging to individual censored patients, distributed as follows: Adrenocortical Carcinoma (ACC, *n* = 93), Bladder Urothelial Carcinoma (BLCA, *n* = 411), Breast Invasive Carcinoma (BRCA, *n* = 1108), Cervical Squamous Cell Carcinoma (CESC, *n* = 310), Cholangiocarcinoma (CHOL, *n* = 51), Colorectal Adenocarcinoma (COADREAD, *n* = 640), Diffuse Large B-Cell Lymphoma (DLBC, *n* = 48), Esophageal Carcinoma (ESCA, *n* = 186), Glioblastoma Multiforme (GBM, *n* = 615), Head and Neck Squamous Cell Carcinoma (HNSC, *n* = 530), Kidney Chromophobe (KICH, *n* = 66), Kidney Renal Clear Cell Carcinoma (KIRC, *n* = 538), Kidney Renal Papillary Cell Carcinoma (KIRP, *n* = 293), Acute Myeloid Leukemia (LAML, *n* = 200), Brain Lower Grade Glioma (LGG, *n* = 530), Liver Hepatocellular Carcinoma (LIHC, *n* = 442), Lung Adenocarcinoma (LUAD, *n* = 588), Lung Squamous Cell Carcinoma (LUSC, *n* = 511), Mesothelioma (MESO, *n* = 87), Ovarian Serous Cystadenocarcinoma (OV, *n* = 609), Pancreatic Adenocarcinoma (PAAD, *n* = 186), Pheochromocytoma and Paraganglioma (PCPG, *n* = 178), Prostate Adenocarcinoma (PRAD, *n* = 499), Sarcoma (SARC, *n* = 265), Skin Cutaneous Melanoma (SKCM, *n* = 480), Stomach Adenocarcinoma (STAD, *n* = 478), Testicular Germ Cell Cancer (TGCT, *n* = 156), Thyroid Carcinoma (THCA, *n* = 516), Thymoma (THYM, *n* = 124), Uterine Corpus Endometrial Carcinoma (UCEC, *n* = 549), Uterine Carcinosarcoma (UCS, *n* = 57), Uveal Melanoma (UVM, *n* = 80). For each sample/patient, the median expression values of the investigated signatures were calculated. According to these values, patients were divided in two groups characterized by high or low expression of the signatures. The Kaplan-Meier survival analysis was performed by using the survival package in R [81].

### 4.5. Estimation of the Contribution/Perturbation of the Immune Infiltration to the Survival Analysis 

The infiltration of immune cells in the tumor biopsies was evaluated by using MCP counter method [44]. Briefly, immunological signatures were retrieved by using the R package MCPcounter. The previously described dataset of 11,424 samples was interrogated with these signatures [44] and each sample was associated to the median value of expression of each signature. According to these values, patients were segregated in two groups and the Kaplan-Meier method was applied to calculate the survival rate.

To evaluate the contribution/disturbance of the inflammatory infiltrate to the prediction of survival based on the transformation signatures, patients were divided into four groups accordingly to the expression levels of genes belonging to the MCPcounter signatures and to the transformation signatures: High-high (high levels of both), high-low (high MCP/low transformation), low-low (low levels of both), or low-high (low MCP-high transformation).

The ‘survfit’ function and the ‘survdiff’ function were used to generate the Kaplan-Meier curves and to calculate the significance.

## Figures and Tables

**Table 1:** List of the common genes and of the relative signatures modulated during the in vitro transformation process.

GENE	SIGNATURE	HALLMARK
***DOCK4***	Upregulated	Complement/Spindle
***G0S2***	Upregulated	TNF-NFKB/KRAS
***SRPX***	Upregulated	Hypoxia
***CDH11***	Downregulated A	EMT/APICAL
***DKK1***	Downregulated A	EMT
***GREM1***	Downregulated A	EMT
***MYLK***	Downregulated A	Adipogenesis/Myogenesis/EMT
***SPRY2***	Downregulated A	KRAS
***ARID5B***	Downregulated B	Androgen/IFNγ
***DUSP4***	Downregulated B	TNFα-NFKB
***ELF1***	Downregulated B	IFNα/ESTROGEN
***LPAR1***	Downregulated B	Inflammation/UV
***MX1***	Downregulated B	IFNα/IFNγ
***SOCS2***	Downregulated B	IL2-STAT5
***TNFRSF11B***	Downregulated B	EMT/APICAL

## Supplementary Materials

The following are available online at https://www.mdpi.com/1422-0067/20/24/6283/s1.
