# Supplementary material for: Genetic Programs Driving Oncogenic Transformation: Lessons from In Vitro Models
Source: Int J Mol Sci. 2019 Dec 12;20(24):6283. doi: 10.3390/ijms20246283 (PMC6940909; doi:10.3390/ijms20246283)
Supplement: Supplementary file 1 [file ijms-20-06283-s001.zip › supplemental submitted/supplemental submitted/supplemental submitted/Supplementary figures legends.pdf]

SUPPLEMENTARY MATERIALS

Genetic programs driving the oncogenic transformation: lessons from *in vitro* models

Eros Di Giorgio[1], Harikrishnareddy Paluvai[1], Raffaella Picco[1], Claudio Brancolini[1,2]

[1]Department of Medicine, Università degli Studi di Udine. P.le Kolbe 4 - 33100 Udine Italy.
[2]Address correspondence to: Claudio Brancolini claudio.brancolini@uniud.it

Figure S1. Effect of high levels of *DOCK4* expression on the survival rate in different cancer types. TCGA survival data analysis on tumors grouped for high levels of *DOCK4* expression alone (>the third quartile/high expressing) compared to all other cases (< the third quartile/low expressing). Cases were: colorectal adenocarcinoma (all n=382, high expressing n=95), kidney chromophobe (all n=65, high expressing n=16), brain low grade glioma (all n= 514, high expressing n=128), stomach adenocarcinoma (all n=412, high expressing n=103), uveal melanoma (all n=80, high expressing n=20), mesothelioma (all n=87, high expressing n=21) and skin cutaneous melanoma (all n=443, high expressing n=110).

Figure S2. Effect of high levels of *SRPX* expression on the survival rate in different cancer types. TCGA survival data analysis on tumors grouped for high levels of *SRPX* expression alone (>the third quartile/high expressing) compared to all other cases (< the third quartile/low expressing). Cases were: bladder urothelial carcinoma (all n=407, high expressing n=101), colorectal adenocarcinoma (all n=592, high expressing n=148), head and neck squamous cell carcinoma (all n=515, high expressing n=128), kidney renal clear cell carcinoma (all n=510, high expressing n=127), kidney renal papillary cell carcinoma (all n= 283 high expressing n=70), thyroid carcinoma (all n=498, high expressing n=124), uterine corpus endometrial carcinoma (all n=527, high expressing n=131) and skin cutaneous melanoma (all n=443, high expressing n=110).

Figure S3. The contribution of infiltrating immune/inflammatory cells to cancer survival. Kaplan-Meier survival analysis related to the signatures marking the infiltration of different immune/inflammatory cells. TCGA survival data analysis on tumors grouped respect to a median expression levels of a signature marking a specific immune cells population, as indicated. High levels > the median). Cases: A) Sarcoma (all=253, high=126) and skin cutaneous melanoma (all=443, high=221). B) Brain low grade glioma (all=514, high=257) and skin cutaneous melanoma (all=443, high=221). C) Sarcoma (all=253, high=126) and skin cutaneous melanoma (all=443, high=221). D) Brain low grade glioma (all=514, high=257), sarcoma (all=253, high=126) and skin cutaneous melanoma (all=443, high=221). E) Brain low grade glioma (all=514, high=257) and skin cutaneous melanoma (all=443, high=221).

Figure S4. The contribution of infiltrating immune/inflammatory cells to cancer survival. Kaplan-Meier survival analysis related to the signatures marking the infiltration of different immune/inflammatory cells. TCGA survival data analysis on tumors grouped respect to a median expression levels of a signature marking a specific immune cells population, as indicated. High levels > the median). Cases: A) Kidney renal clear cell carcinoma (all=510, high=255), sarcoma (all=253, high=126) and skin cutaneous melanoma (all=443, high=221). B) Brain low grade glioma (all=514, high=257). C) Skin cutaneous melanoma (all=443, high=221).
