# Supplementary material for: Genetic Programs Driving Oncogenic Transformation: Lessons from In Vitro Models
Source: Int J Mol Sci. 2019 Dec 12;20(24):6283. doi: 10.3390/ijms20246283 (PMC6940909; doi:10.3390/ijms20246283)
Supplement: Supplementary file 1 [file ijms-20-06283-s001.zip › supplemental submitted/supplemental submitted/supplemental submitted/Table SIA.docx]

**Table SIA. Hallmarks up-regulated by RAS**

| Hallmarks gene sets | Genes in Gene Set | Genes in overlap | % of overlap | p-value | FDR q-value |
| --- | --- | --- | --- | --- | --- |
| HALLMARK_TNFA_SIGNALING_VIA_NFKB | 200 | 39 | 19.5 | 6.6E-41 | 3.3E-39 |
| HALLMARK_HYPOXIA | 200 | 30 | 15 | 4.13E-28 | 1.03E-26 |
| HALLMARK_MTORC1_SIGNALING | 200 | 28 | 14 | 1.87E-25 | 2.34E-24 |
| HALLMARK_P53_PATHWAY | 200 | 28 | 14 | 1.87E-25 | 2.34E-24 |
| HALLMARK_GLYCOLYSIS | 200 | 24 | 12 | 2.25E-20 | 2.25E-19 |
| HALLMARK_EPITHELIAL_MESENCHYMAL_TRANSITION | 200 | 23 | 11.5 | 3.73E-19 | 3.11E-18 |
| HALLMARK_APOPTOSIS | 161 | 19 | 11.8 | 2.92E-16 | 2.08E-15 |
| HALLMARK_UV_RESPONSE_UP | 158 | 18 | 11.39 | 3.34E-15 | 2.09E-14 |
| HALLMARK_COMPLEMENT | 200 | 19 | 9.5 | 1.65E-14 | 9.19E-14 |
| HALLMARK_INFLAMMATORY_RESPONSE | 200 | 18 | 9 | 2.08E-13 | 9.47E-13 |
| HALLMARK_INTERFERON_GAMMA_RESPONSE | 200 | 18 | 9 | 2.08E-13 | 9.47E-13 |
| HALLMARK_CHOLESTEROL_HOMEOSTASIS | 74 | 10 | 13.51 | 8.87E-10 | 3.69E-09 |
| HALLMARK_ESTROGEN_RESPONSE_EARLY | 200 | 14 | 7 | 2.74E-09 | 1.06E-08 |
| HALLMARK_MYOGENESIS | 200 | 13 | 6.5 | 2.46E-08 | 8.77E-08 |
| HALLMARK_COAGULATION | 138 | 11 | 7.97 | 3.67E-08 | 0.000000122 |
| HALLMARK_ESTROGEN_RESPONSE_LATE | 200 | 12 | 6 | 0.000000203 | 0.000000634 |
| HALLMARK_UNFOLDED_PROTEIN_RESPONSE | 113 | 9 | 7.96 | 0.000000649 | 0.00000191 |
| HALLMARK_ADIPOGENESIS | 200 | 11 | 5.5 | 0.00000154 | 0.00000404 |
| HALLMARK_ALLOGRAFT_REJECTION | 200 | 11 | 5.5 | 0.00000154 | 0.00000404 |
| HALLMARK_UV_RESPONSE_DN | 144 | 9 | 6.25 | 0.00000489 | 0.0000122 |
| HALLMARK_HEME_METABOLISM | 200 | 10 | 5 | 0.0000106 | 0.0000225 |
| HALLMARK_IL2_STAT5_SIGNALING | 200 | 10 | 5 | 0.0000106 | 0.0000225 |
| HALLMARK_KRAS_SIGNALING_UP | 200 | 10 | 5 | 0.0000106 | 0.0000225 |
| HALLMARK_IL6_JAK_STAT3_SIGNALING | 87 | 7 | 8.05 | 0.0000108 | 0.0000225 |
| HALLMARK_ANDROGEN_RESPONSE | 101 | 7 | 6.93 | 0.0000287 | 0.0000574 |
| HALLMARK_XENOBIOTIC_METABOLISM | 200 | 9 | 4.5 | 0.0000663 | 0.000128 |
| HALLMARK_APICAL_JUNCTION | 200 | 8 | 4 | 0.000372 | 0.000688 |
| HALLMARK_BILE_ACID_METABOLISM | 112 | 6 | 5.36 | 0.000437 | 0.000781 |
| HALLMARK_REACTIVE_OXIGEN_SPECIES_PATHWAY | 49 | 4 | 8.16 | 0.000847 | 0.00146 |
| HALLMARK_E2F_TARGETS | 200 | 7 | 3.5 | 0.00185 | 0.00308 |
| HALLMARK_PI3K_AKT_MTOR_SIGNALING | 105 | 5 | 4.76 | 0.00221 | 0.00357 |
| HALLMARK_FATTY_ACID_METABOLISM | 158 | 6 | 3.8 | 0.00258 | 0.00403 |
| HALLMARK_SPERMATOGENESIS | 135 | 5 | 3.7 | 0.00644 | 0.00977 |
| HALLMARK_DNA_REPAIR | 150 | 5 | 3.33 | 0.00991 | 0.0146 |
| HALLMARK_MITOTIC_SPINDLE | 200 | 5 | 2.5 | 0.0302 | 0.0431 |
