# Supplementary material for: Genetic Programs Driving Oncogenic Transformation: Lessons from In Vitro Models
Source: Int J Mol Sci. 2019 Dec 12;20(24):6283. doi: 10.3390/ijms20246283 (PMC6940909; doi:10.3390/ijms20246283)
Supplement: Supplementary file 1 [file ijms-20-06283-s001.zip › supplemental submitted/supplemental submitted/supplemental submitted/Table SIB.docx]

**Table SIB** **Hallmarks up-regulated by MYC**

| Hallmarks gene sets | Genes in Gene Set | Genes in overlap | % of overlap | p-value | FDR q-value |
| --- | --- | --- | --- | --- | --- |
| HALLMARK_MYC_TARGETS_V2 | 58 | 11 | 18.97 | 2.16E-11 | 1.08E-09 |
| HALLMARK_ESTROGEN_RESPONSE_EARLY | 200 | 17 | 8.5 | 5.19E-11 | 1.3E-09 |
| HALLMARK_IL2_STAT5_SIGNALING | 200 | 16 | 8 | 4.71E-10 | 7.85E-09 |
| HALLMARK_ESTROGEN_RESPONSE_LATE | 200 | 14 | 7 | 3.15E-08 | 0.000000315 |
| HALLMARK_MYC_TARGETS_V1 | 200 | 14 | 7 | 3.15E-08 | 0.000000315 |
| HALLMARK_KRAS_SIGNALING_UP | 200 | 13 | 6.5 | 0.000000231 | 0.00000165 |
| HALLMARK_MTORC1_SIGNALING | 200 | 13 | 6.5 | 0.000000231 | 0.00000165 |
| HALLMARK_P53_PATHWAY | 200 | 12 | 6 | 0.00000156 | 0.00000887 |
| HALLMARK_PEROXISOME | 104 | 9 | 8.65 | 0.0000016 | 0.00000887 |
| HALLMARK_GLYCOLYSIS | 200 | 11 | 5.5 | 0.00000971 | 0.0000486 |
| HALLMARK_CHOLESTEROL_HOMEOSTASIS | 74 | 7 | 9.46 | 0.0000129 | 0.0000589 |
| HALLMARK_ANDROGEN_RESPONSE | 101 | 7 | 6.93 | 0.0000971 | 0.000405 |
| HALLMARK_REACTIVE_OXIGEN_SPECIES_PATHWAY | 49 | 5 | 10.2 | 0.000161 | 0.000617 |
| HALLMARK_BILE_ACID_METABOLISM | 112 | 7 | 6.25 | 0.000185 | 0.000662 |
| HALLMARK_ANGIOGENESIS | 36 | 4 | 11.11 | 0.000535 | 0.00178 |
| HALLMARK_INFLAMMATORY_RESPONSE | 200 | 8 | 4 | 0.00131 | 0.00362 |
| HALLMARK_MYOGENESIS | 200 | 8 | 4 | 0.00131 | 0.00362 |
| HALLMARK_TNFA_SIGNALING_VIA_NFKB | 200 | 8 | 4 | 0.00131 | 0.00362 |
| HALLMARK_FATTY_ACID_METABOLISM | 158 | 7 | 4.43 | 0.00145 | 0.00362 |
| HALLMARK_UV_RESPONSE_UP | 158 | 7 | 4.43 | 0.00145 | 0.00362 |
| HALLMARK_NOTCH_SIGNALING | 32 | 3 | 9.38 | 0.00451 | 0.0107 |
| HALLMARK_ADIPOGENESIS | 200 | 7 | 3.5 | 0.00536 | 0.0116 |
| HALLMARK_OXIDATIVE_PHOSPHORYLATION | 200 | 7 | 3.5 | 0.00536 | 0.0116 |
| HALLMARK_APOPTOSIS | 161 | 6 | 3.73 | 0.0072 | 0.015 |
| HALLMARK_WNT_BETA_CATENIN_SIGNALING | 42 | 3 | 7.14 | 0.00967 | 0.0193 |
| HALLMARK_APICAL_SURFACE | 44 | 3 | 6.82 | 0.011 | 0.0211 |
| HALLMARK_COMPLEMENT | 200 | 6 | 3 | 0.0193 | 0.0311 |
| HALLMARK_G2M_CHECKPOINT | 200 | 6 | 3 | 0.0193 | 0.0311 |
| HALLMARK_HEME_METABOLISM | 200 | 6 | 3 | 0.0193 | 0.0311 |
| HALLMARK_HYPOXIA | 200 | 6 | 3 | 0.0193 | 0.0311 |
| HALLMARK_MITOTIC_SPINDLE | 200 | 6 | 3 | 0.0193 | 0.0311 |
