# Supplementary material for: Genetic Programs Driving Oncogenic Transformation: Lessons from In Vitro Models
Source: Int J Mol Sci. 2019 Dec 12;20(24):6283. doi: 10.3390/ijms20246283 (PMC6940909; doi:10.3390/ijms20246283)
Supplement: Supplementary file 1 [file ijms-20-06283-s001.zip › supplemental submitted/supplemental submitted/supplemental submitted/Table SIC.docx]

**Table SIC. Hallmarks up-regulated by HDAC4**

| Hallmarks gene sets | Genes in Gene set | Genes in overlap | % of overlap | p-value | FDR  q-value |
| --- | --- | --- | --- | --- | --- |
| HALLMARK_KRAS_SIGNALING_UP | 200 | 20 | 10 | 8.81E-14 | 4.4E-12 |
| HALLMARK_EPITHELIAL_MESENCHYMAL_TRANSITION | 200 | 17 | 8.5 | 8.6E-11 | 2.15E-09 |
| HALLMARK_TNFA_SIGNALING_VIA_NFKB | 200 | 16 | 8 | 7.54E-10 | 1.26E-08 |
| HALLMARK_INFLAMMATORY_RESPONSE | 200 | 15 | 7.5 | 6.18E-09 | 7.73E-08 |
| HALLMARK_XENOBIOTIC_METABOLISM | 200 | 11 | 5.5 | 0.0000132 | 0.000132 |
| HALLMARK_COMPLEMENT | 200 | 10 | 5 | 0.0000722 | 0.000401 |
| HALLMARK_ESTROGEN_RESPONSE_EARLY | 200 | 10 | 5 | 0.0000722 | 0.000401 |
| HALLMARK_HYPOXIA | 200 | 10 | 5 | 0.0000722 | 0.000401 |
| HALLMARK_MITOTIC_SPINDLE | 200 | 10 | 5 | 0.0000722 | 0.000401 |
| HALLMARK_ALLOGRAFT_REJECTION | 200 | 9 | 4.5 | 0.000359 | 0.00138 |
| HALLMARK_ESTROGEN_RESPONSE_LATE | 200 | 9 | 4.5 | 0.000359 | 0.00138 |
| HALLMARK_HEME_METABOLISM | 200 | 9 | 4.5 | 0.000359 | 0.00138 |
| HALLMARK_INTERFERON_GAMMA_RESPONSE | 200 | 9 | 4.5 | 0.000359 | 0.00138 |
| HALLMARK_COAGULATION | 138 | 7 | 5.07 | 0.000796 | 0.00284 |
| HALLMARK_UV_RESPONSE_DN | 144 | 7 | 4.86 | 0.00102 | 0.00341 |
| HALLMARK_APICAL_SURFACE | 44 | 4 | 9.09 | 0.0013 | 0.00407 |
| HALLMARK_ADIPOGENESIS | 200 | 8 | 4 | 0.0016 | 0.00445 |
| HALLMARK_APICAL_JUNCTION | 200 | 8 | 4 | 0.0016 | 0.00445 |
| HALLMARK_PROTEIN_SECRETION | 96 | 5 | 5.21 | 0.00396 | 0.0104 |
| HALLMARK_GLYCOLYSIS | 200 | 7 | 3.5 | 0.00637 | 0.0157 |
| HALLMARK_ANGIOGENESIS | 36 | 3 | 8.33 | 0.00689 | 0.0157 |
| HALLMARK_HEDGEHOG_SIGNALING | 36 | 3 | 8.33 | 0.00689 | 0.0157 |
| HALLMARK_BILE_ACID_METABOLISM | 112 | 5 | 4.46 | 0.00755 | 0.0164 |
| HALLMARK_IL6_JAK_STAT3_SIGNALING | 87 | 4 | 4.6 | 0.0149 | 0.031 |
| HALLMARK_G2M_CHECKPOINT | 200 | 6 | 3 | 0.0222 | 0.0411 |
| HALLMARK_MTORC1_SIGNALING | 200 | 6 | 3 | 0.0222 | 0.0411 |
| HALLMARK_P53_PATHWAY | 200 | 6 | 3 | 0.0222 | 0.0411 |
| HALLMARK_MYC_TARGETS_V2 | 58 | 3 | 5.17 | 0.025 | 0.0447 |
