# Supplementary material for: Genetic Programs Driving Oncogenic Transformation: Lessons from In Vitro Models
Source: Int J Mol Sci. 2019 Dec 12;20(24):6283. doi: 10.3390/ijms20246283 (PMC6940909; doi:10.3390/ijms20246283)
Supplement: Supplementary file 1 [file ijms-20-06283-s001.zip › supplemental submitted/supplemental submitted/supplemental submitted/Table SIIA.docx]

**Table SIIA. Hallmarks down-regulated by RAS**

| Hallmarks gene sets | Genes in Gene set | Genes in overlap | % of overlap | p-value | FDR  q-value |
| --- | --- | --- | --- | --- | --- |
| HALLMARK_EPITHELIAL_MESENCHYMAL_TRANSITION | 200 | 32 | 16 | 1.47E-32 | 7.35E-31 |
| HALLMARK_INTERFERON_ALPHA_RESPONSE | 97 | 25 | 25.77 | 2.86E-31 | 7.16E-30 |
| HALLMARK_INTERFERON_GAMMA_RESPONSE | 200 | 29 | 14.5 | 2.56E-28 | 4.27E-27 |
| HALLMARK_UV_RESPONSE_DN | 144 | 22 | 15.28 | 2.77E-22 | 3.47E-21 |
| HALLMARK_TNFA_SIGNALING_VIA_NFKB | 200 | 18 | 9 | 2.55E-14 | 2.55E-13 |
| HALLMARK_ESTROGEN_RESPONSE_EARLY | 200 | 17 | 8.5 | 3.43E-13 | 2.86E-12 |
| HALLMARK_ESTROGEN_RESPONSE_LATE | 200 | 14 | 7 | 5.62E-10 | 4.01E-09 |
| HALLMARK_APOPTOSIS | 161 | 12 | 7.45 | 4.76E-09 | 2.73E-08 |
| HALLMARK_INFLAMMATORY_RESPONSE | 200 | 13 | 6.5 | 5.72E-09 | 2.73E-08 |
| HALLMARK_KRAS_SIGNALING_UP | 200 | 13 | 6.5 | 5.72E-09 | 2.73E-08 |
| HALLMARK_ANDROGEN_RESPONSE | 101 | 10 | 9.9 | 6.01E-09 | 2.73E-08 |
| HALLMARK_TGF_BETA_SIGNALING | 54 | 8 | 14.81 | 8.01E-09 | 3.34E-08 |
| HALLMARK_APICAL_JUNCTION | 200 | 12 | 6 | 5.36E-08 | 0.000000191 |
| HALLMARK_MYOGENESIS | 200 | 12 | 6 | 5.36E-08 | 0.000000191 |
| HALLMARK_G2M_CHECKPOINT | 200 | 9 | 4.5 | 0.0000256 | 0.0000853 |
| HALLMARK_COMPLEMENT | 200 | 8 | 4 | 0.000162 | 0.000477 |
| HALLMARK_HYPOXIA | 200 | 8 | 4 | 0.000162 | 0.000477 |
| HALLMARK_COAGULATION | 138 | 6 | 4.35 | 0.000692 | 0.00192 |
| HALLMARK_NOTCH_SIGNALING | 32 | 3 | 9.38 | 0.00184 | 0.00485 |
| HALLMARK_WNT_BETA_CATENIN_SIGNALING | 42 | 3 | 7.14 | 0.00404 | 0.00863 |
| HALLMARK_ALLOGRAFT_REJECTION | 200 | 6 | 3 | 0.00449 | 0.00863 |
| HALLMARK_HEME_METABOLISM | 200 | 6 | 3 | 0.00449 | 0.00863 |
| HALLMARK_IL2_STAT5_SIGNALING | 200 | 6 | 3 | 0.00449 | 0.00863 |
| HALLMARK_KRAS_SIGNALING_DN | 200 | 6 | 3 | 0.00449 | 0.00863 |
| HALLMARK_MITOTIC_SPINDLE | 200 | 6 | 3 | 0.00449 | 0.00863 |
| HALLMARK_P53_PATHWAY | 200 | 6 | 3 | 0.00449 | 0.00863 |
| HALLMARK_ADIPOGENESIS | 200 | 5 | 2.5 | 0.019 | 0.0318 |
| HALLMARK_E2F_TARGETS | 200 | 5 | 2.5 | 0.019 | 0.0318 |
| HALLMARK_XENOBIOTIC_METABOLISM | 200 | 5 | 2.5 | 0.019 | 0.0318 |
| HALLMARK_CHOLESTEROL_HOMEOSTASIS | 74 | 3 | 4.05 | 0.0191 | 0.0318 |
| HALLMARK_SPERMATOGENESIS | 135 | 4 | 2.96 | 0.0201 | 0.0324 |
| HALLMARK_ANGIOGENESIS | 36 | 2 | 5.56 | 0.0307 | 0.048 |
