# Supplementary material for: Genetic Programs Driving Oncogenic Transformation: Lessons from In Vitro Models
Source: Int J Mol Sci. 2019 Dec 12;20(24):6283. doi: 10.3390/ijms20246283 (PMC6940909; doi:10.3390/ijms20246283)
Supplement: Supplementary file 1 [file ijms-20-06283-s001.zip › supplemental submitted/supplemental submitted/supplemental submitted/table SIIB.docx]

| Hallmarks gene sets | Genes in Gene set | Genes in overlap | % of overlap | p-value | FDR  q-value |
| --- | --- | --- | --- | --- | --- |
| HALLMARK_INTERFERON_GAMMA_RESPONSE | 200 | 57 | 28.5 | 7.18E-59 | 3.59E-57 |
| HALLMARK_INTERFERON_ALPHA_RESPONSE | 97 | 43 | 44.33 | 1.67E-54 | 4.18E-53 |
| HALLMARK_UV_RESPONSE_DN | 144 | 24 | 16.67 | 1.8E-19 | 3E-18 |
| HALLMARK_EPITHELIAL_MESENCHYMAL_TRANSITION | 200 | 25 | 12.5 | 3.95E-17 | 4.93E-16 |
| HALLMARK_TNFA_SIGNALING_VIA_NFKB | 200 | 21 | 10.5 | 4.28E-13 | 4.28E-12 |
| HALLMARK_KRAS_SIGNALING_UP | 200 | 18 | 9 | 2.61E-10 | 2.18E-09 |
| HALLMARK_INFLAMMATORY_RESPONSE | 200 | 16 | 8 | 0.000000014 | 9.97E-08 |
| HALLMARK_ESTROGEN_RESPONSE_EARLY | 200 | 15 | 7.5 | 9.25E-08 | 0.000000514 |
| HALLMARK_IL2_STAT5_SIGNALING | 200 | 15 | 7.5 | 9.25E-08 | 0.000000514 |
| HALLMARK_P53_PATHWAY | 200 | 14 | 7 | 0.000000572 | 0.00000286 |
| HALLMARK_IL6_JAK_STAT3_SIGNALING | 87 | 9 | 10.34 | 0.0000025 | 0.0000114 |
| HALLMARK_COAGULATION | 138 | 10 | 7.25 | 0.0000173 | 0.0000669 |
| HALLMARK_COMPLEMENT | 200 | 12 | 6 | 0.0000174 | 0.0000669 |
| HALLMARK_PROTEIN_SECRETION | 96 | 8 | 8.33 | 0.0000443 | 0.000158 |
| HALLMARK_CHOLESTEROL_HOMEOSTASIS | 74 | 7 | 9.46 | 0.0000591 | 0.000197 |
| HALLMARK_ALLOGRAFT_REJECTION | 200 | 11 | 5.5 | 0.0000849 | 0.000223 |
| HALLMARK_ESTROGEN_RESPONSE_LATE | 200 | 11 | 5.5 | 0.0000849 | 0.000223 |
| HALLMARK_KRAS_SIGNALING_DN | 200 | 11 | 5.5 | 0.0000849 | 0.000223 |
| HALLMARK_MYOGENESIS | 200 | 11 | 5.5 | 0.0000849 | 0.000223 |
| HALLMARK_APOPTOSIS | 161 | 9 | 5.59 | 0.000327 | 0.000817 |
| HALLMARK_HEME_METABOLISM | 200 | 10 | 5 | 0.000379 | 0.00086 |
| HALLMARK_XENOBIOTIC_METABOLISM | 200 | 10 | 5 | 0.000379 | 0.00086 |
| HALLMARK_APICAL_JUNCTION | 200 | 9 | 4.5 | 0.00153 | 0.00306 |
| HALLMARK_GLYCOLYSIS | 200 | 9 | 4.5 | 0.00153 | 0.00306 |
| HALLMARK_HYPOXIA | 200 | 9 | 4.5 | 0.00153 | 0.00306 |
| HALLMARK_ANDROGEN_RESPONSE | 101 | 6 | 5.94 | 0.00235 | 0.00452 |
| HALLMARK_PI3K_AKT_MTOR_SIGNALING | 105 | 6 | 5.71 | 0.00286 | 0.00529 |
| HALLMARK_ADIPOGENESIS | 200 | 8 | 4 | 0.00558 | 0.00996 |
| HALLMARK_HEDGEHOG_SIGNALING | 36 | 3 | 8.33 | 0.0121 | 0.0208 |

Table SIIB. Hallmarks down-regulated by MYC
