# Supplementary material for: Genetic Programs Driving Oncogenic Transformation: Lessons from In Vitro Models
Source: Int J Mol Sci. 2019 Dec 12;20(24):6283. doi: 10.3390/ijms20246283 (PMC6940909; doi:10.3390/ijms20246283)
Supplement: Supplementary file 1 [file ijms-20-06283-s001.zip › supplemental submitted/supplemental submitted/supplemental submitted/Table SIIC.docx]

| Hallmarks gene sets | Genes in Gene set | Genes in overlap | % of overlap | p-value | FDR  q-value |
| --- | --- | --- | --- | --- | --- |
| HALLMARK_EPITHELIAL_MESENCHYMAL_TRANSITION | 200 | 50 | 25 | 7.89E-41 | 3.94E-39 |
| HALLMARK_HYPOXIA | 200 | 34 | 17 | 2.66E-22 | 6.65E-21 |
| HALLMARK_INTERFERON_GAMMA_RESPONSE | 200 | 32 | 16 | 3.04E-20 | 5.07E-19 |
| HALLMARK_UV_RESPONSE_DN | 144 | 26 | 18.06 | 4.69E-18 | 5.87E-17 |
| HALLMARK_MYOGENESIS | 200 | 29 | 14.5 | 2.79E-17 | 2.79E-16 |
| HALLMARK_IL2_STAT5_SIGNALING | 200 | 25 | 12.5 | 1.4E-13 | 1.17E-12 |
| HALLMARK_INTERFERON_ALPHA_RESPONSE | 97 | 17 | 17.53 | 4.52E-12 | 3.23E-11 |
| HALLMARK_ESTROGEN_RESPONSE_EARLY | 200 | 23 | 11.5 | 7.6E-12 | 4.22E-11 |
| HALLMARK_P53_PATHWAY | 200 | 23 | 11.5 | 7.6E-12 | 4.22E-11 |
| HALLMARK_APICAL_JUNCTION | 200 | 21 | 10.5 | 3.39E-10 | 1.41E-09 |
| HALLMARK_INFLAMMATORY_RESPONSE | 200 | 21 | 10.5 | 3.39E-10 | 1.41E-09 |
| HALLMARK_TNFA_SIGNALING_VIA_NFKB | 200 | 21 | 10.5 | 3.39E-10 | 1.41E-09 |
| HALLMARK_APOPTOSIS | 161 | 17 | 10.56 | 1.48E-08 | 5.69E-08 |
| HALLMARK_ALLOGRAFT_REJECTION | 200 | 18 | 9 | 6.78E-08 | 0.000000212 |
| HALLMARK_COMPLEMENT | 200 | 18 | 9 | 6.78E-08 | 0.000000212 |
| HALLMARK_MITOTIC_SPINDLE | 200 | 18 | 9 | 6.78E-08 | 0.000000212 |
| HALLMARK_COAGULATION | 138 | 14 | 10.14 | 0.000000449 | 0.00000132 |
| HALLMARK_CHOLESTEROL_HOMEOSTASIS | 74 | 10 | 13.51 | 0.00000144 | 0.00000399 |
| HALLMARK_ESTROGEN_RESPONSE_LATE | 200 | 16 | 8 | 0.00000173 | 0.00000433 |
| HALLMARK_KRAS_SIGNALING_UP | 200 | 16 | 8 | 0.00000173 | 0.00000433 |
| HALLMARK_GLYCOLYSIS | 200 | 14 | 7 | 0.000034 | 0.000081 |
| HALLMARK_ANGIOGENESIS | 36 | 6 | 16.67 | 0.0000564 | 0.000128 |
| HALLMARK_ADIPOGENESIS | 200 | 13 | 6.5 | 0.000136 | 0.000258 |
| HALLMARK_HEME_METABOLISM | 200 | 13 | 6.5 | 0.000136 | 0.000258 |
| HALLMARK_XENOBIOTIC_METABOLISM | 200 | 13 | 6.5 | 0.000136 | 0.000258 |
| HALLMARK_WNT_BETA_CATENIN_SIGNALING | 42 | 6 | 14.29 | 0.000138 | 0.000258 |
| HALLMARK_ANDROGEN_RESPONSE | 101 | 9 | 8.91 | 0.00014 | 0.000258 |
| HALLMARK_PEROXISOME | 104 | 8 | 7.69 | 0.00087 | 0.00155 |
| HALLMARK_FATTY_ACID_METABOLISM | 158 | 10 | 6.33 | 0.000958 | 0.0016 |
| HALLMARK_UV_RESPONSE_UP | 158 | 10 | 6.33 | 0.000958 | 0.0016 |
| HALLMARK_BILE_ACID_METABOLISM | 112 | 8 | 7.14 | 0.00141 | 0.00227 |
| HALLMARK_PROTEIN_SECRETION | 96 | 7 | 7.29 | 0.00245 | 0.00384 |
| HALLMARK_HEDGEHOG_SIGNALING | 36 | 4 | 11.11 | 0.00476 | 0.00721 |
| HALLMARK_MTORC1_SIGNALING | 200 | 10 | 5 | 0.00531 | 0.00781 |
| HALLMARK_IL6_JAK_STAT3_SIGNALING | 87 | 6 | 6.9 | 0.00645 | 0.00922 |
| HALLMARK_APICAL_SURFACE | 44 | 4 | 9.09 | 0.00973 | 0.0135 |

**Table SIIA. Hallmarks down-regulated by HDAC4**
