# Supplementary figures and images for: Genetic Programs Driving Oncogenic Transformation: Lessons from In Vitro Models
Source: Int J Mol Sci. 2019 Dec 12;20(24):6283. doi: 10.3390/ijms20246283 (PMC6940909; doi:10.3390/ijms20246283)

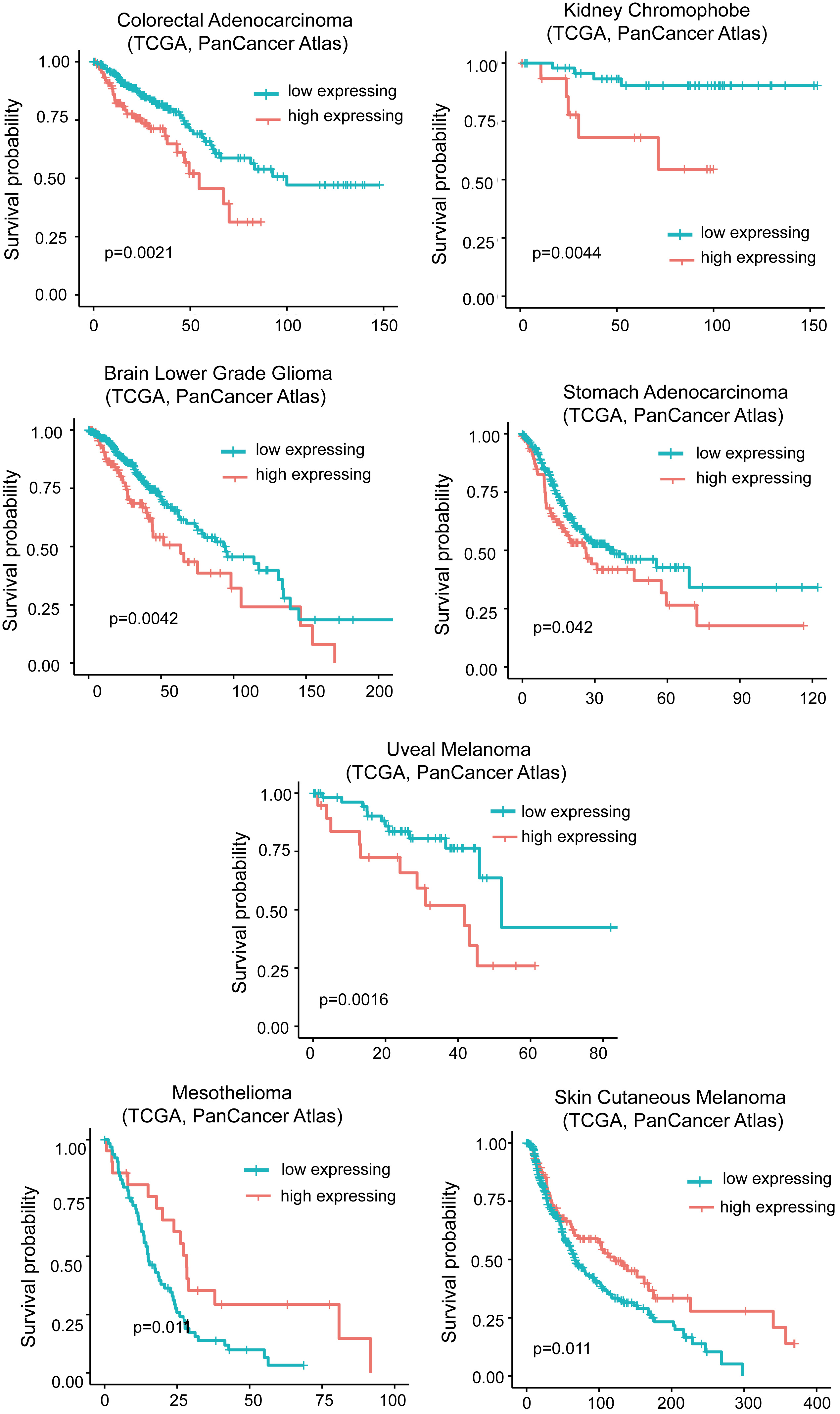

Supplement: Supplementary file 1 [file ijms-20-06283-s001.zip › supplemental submitted/FIGS1.jpg]

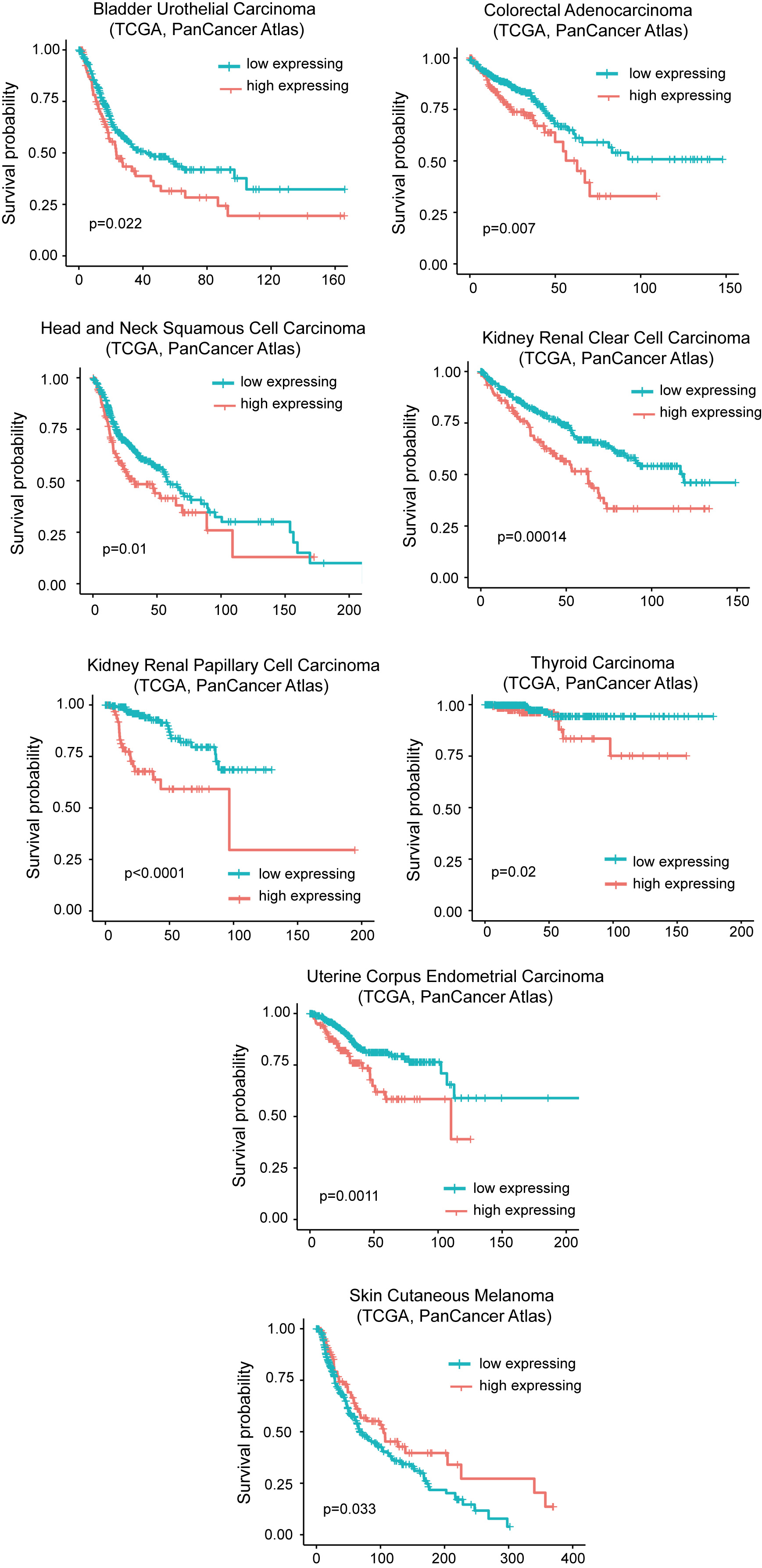

Supplement: Supplementary file 1 [file ijms-20-06283-s001.zip › supplemental submitted/FIGS2.jpg]

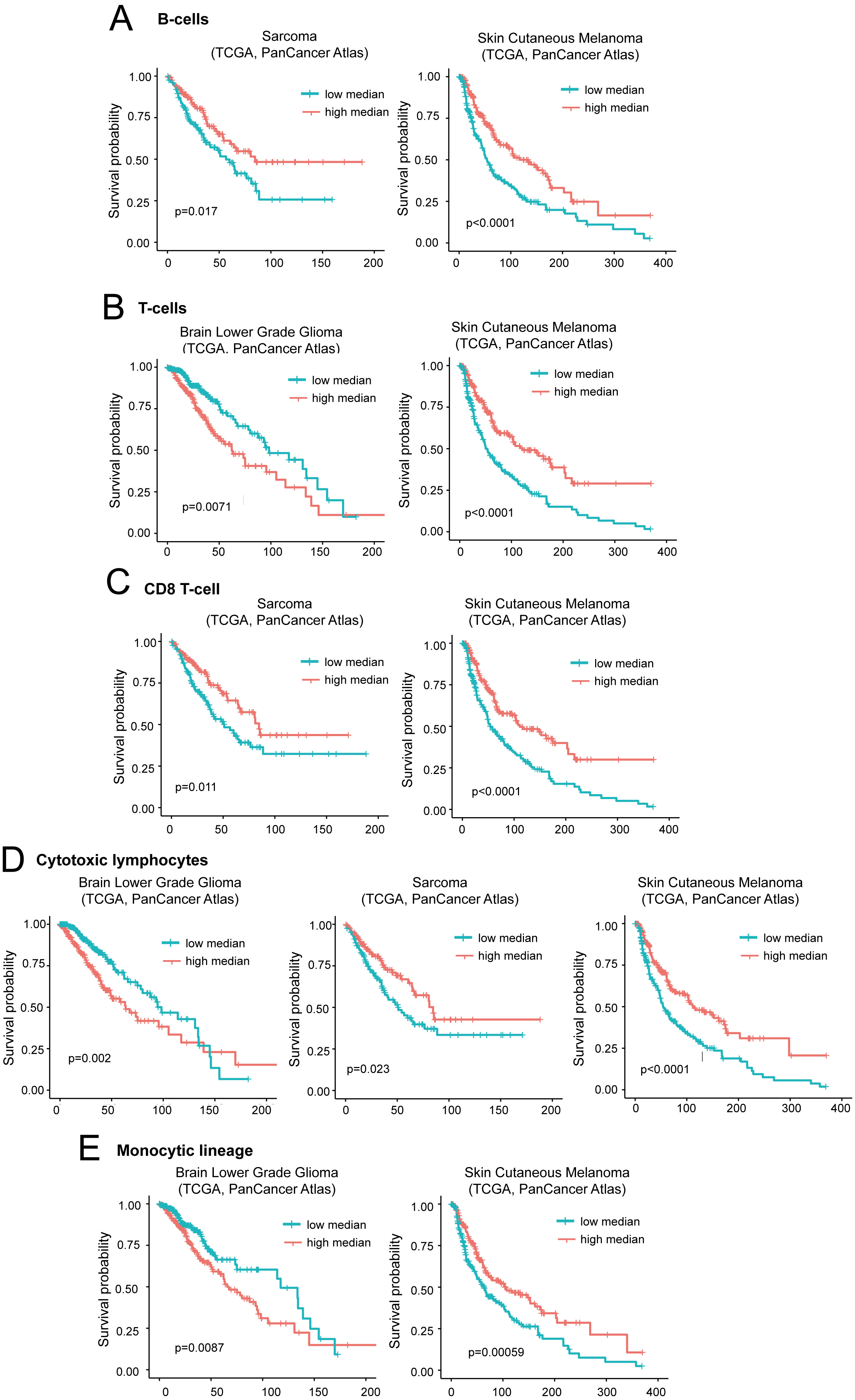

Supplement: Supplementary file 1 [file ijms-20-06283-s001.zip › supplemental submitted/FIGS3.jpg]

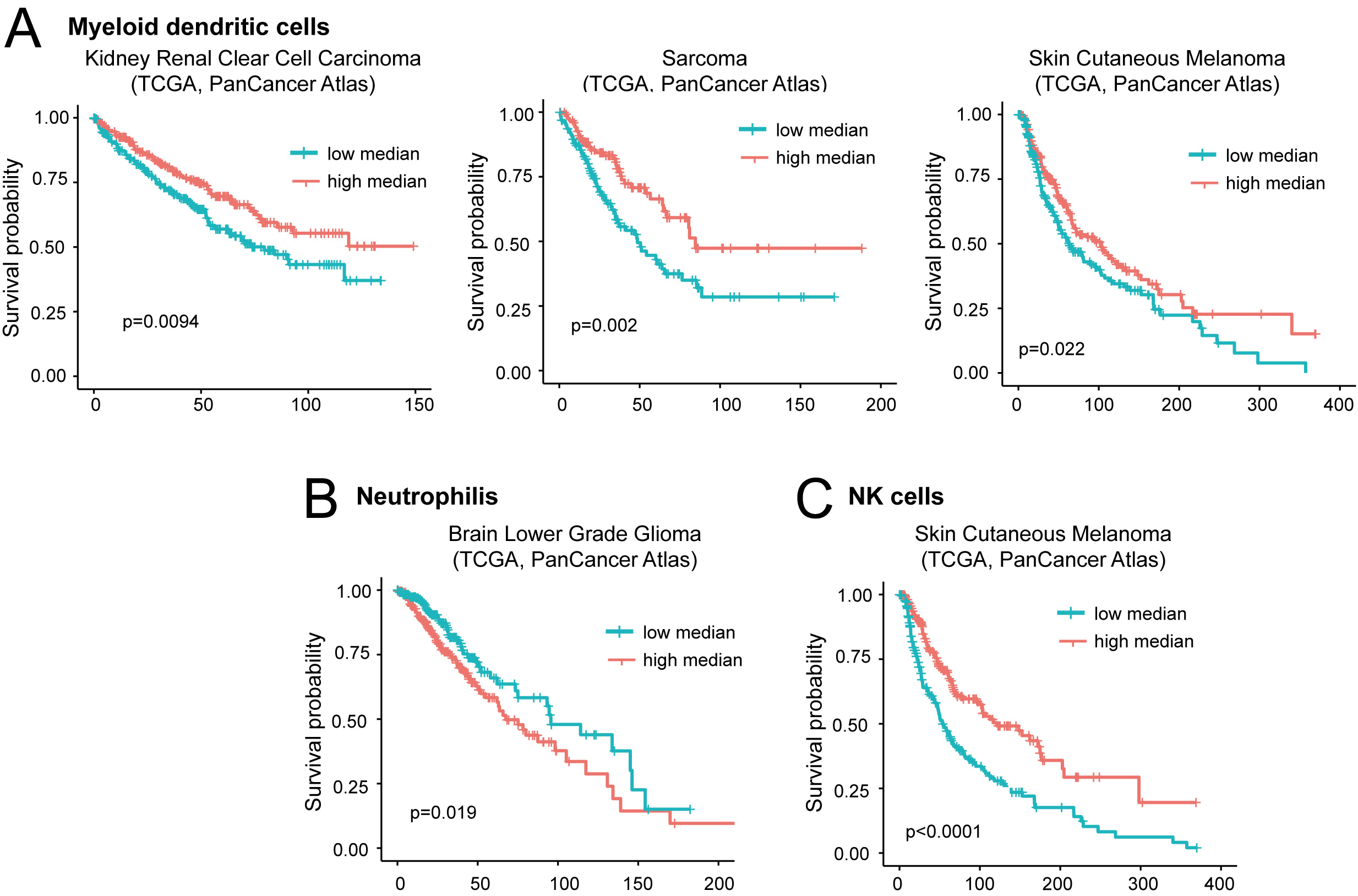

Supplement: Supplementary file 1 [file ijms-20-06283-s001.zip › supplemental submitted/figS4.jpg]
